# Impairment in quality of life with different sport orthopaedic musculoskeletal injuries—A comprehensive analysis of the German arthroscopy register

**DOI:** 10.1002/ksa.70205

**Published:** 2025-11-18

**Authors:** Dominik Szymski, Christoph Lutter, Sina Graeber, Alexander‐Stephan Henze, Maximilian Hinz, Anja Hirschmueller, Thomas Niethammer, Ralf Mueller Rath, Thomas Tischer

**Affiliations:** ^1^ Department of Trauma Surgery University Medical Centre Regensburg Regensburg Germany; ^2^ FIFA Medical Centre of Excellence University Medical Centre Regensburg Regensburg Germany; ^3^ Department of Orthopaedics Rostock University Medical Center Rostock Germany; ^4^ Sports and Rehabilitation Medicine University Hospital Ulm Ulm Germany; ^5^ Center for Musculoskeletal Surgery, Charité – Universitätsmedizin Berlin Corporate Member of Freie Universität Berlin and Humboldt‐Universität zu Berlin Berlin Germany; ^6^ ALTIUS Swiss Sportmed Center AG Rheinfelden Switzerland; ^7^ Department of Orthopaedics and Trauma Surgery Musculoskeletal University Center Munich (MUM), LMU University Hospital, LMU Munich Germany; ^8^ OPND Orthopädische Praxisklinik Neuss and Düsseldorf Germany; ^9^ Department of Orthopaedic and Traumatologic Surgery Waldkrankenhaus Erlangen Germany

**Keywords:** orthopaedic, psychological, registry, sports medicine, trauma

## Abstract

**Purpose:**

The aim of this study was to identify the impairment of quality of life (QoL) for different sport orthopaedic injuries prior to surgery in Germany and build a baseline dataset for these injuries.

**Methods:**

Data from the German arthroscopy registry (DART) between November 2017 and January 2025 were used. EuroQol five‐dimension questionnaire (EQ) value and EQ‐visual analogue scale (EQ‐VAS) were analysed for shoulder, hip, knee and ankle injuries. The EQ index value was calculated using a time‐trade‐off value set for Germany. One‐way analysis of variance (ANOVA) and standardised mean differences (SMD) were calculated to assess between‐group effects.

**Results:**

A total of 9432 patients from the DART were included, predominantly with knee (75.5%) and shoulder (19.1%) pathologies. The highest EQ value was observed in shoulder patients (mean = 0.744 ± 0.22), while the lowest was reported in ankle patients (mean = 0.692 ± 0.26). EQ‐VAS scores were highest in ankle (mean = 66.30 ± 22.1) and lowest in hip patients (mean = 62.41 ± 19.9). One‐way ANOVA revealed significant differences between joint groups (*p* < 0.001). Subgroup analysis showed the highest EQ value in meniscus lesions with future resection (0.806 ± 0.17) and ACL re‐rupture (0.785 ± 0.21), while patella instability (0.633 ± 0.28) and posterior cruciate ligament (PCL) rupture (0.641 ± 0.27) showed the lowest. EQ‐VAS was highest for meniscus lesions with future resection (71.03 ± 19.8) and rotator cuff tear without biceps tendon pathology (68.20 ± 20.8), and lowest for PCL rupture (62.45 ± 18.4) and femoroacetabular impingement (62.20 ± 20.1).

**Conclusion:**

Sport orthopaedic injuries cause a marked reduction in QoL, especially prior to surgical treatment. These findings highlight the need for greater integration of QoL assessment and mental health support in orthopaedic care and research to ensure more holistic, patient‐centred treatment strategies.

**Level of Evidence:**

Level II prognostic prospective cohort study.

**Abbreviations**
ACL: anterior cruciate ligament
AGA: German Society for Arthroscopy and Joint‐Surgery
ANOVA: analysis of variance
BVASK: German society for arthroscopy
DART: German arthroscopy register
DGOU: German Society for Orthopaedics and Trauma
EQ: EuroQol
EQ‐VAS: EuroQoLVisual analogue scale (EQ‐VAS)
GOTS: Society for Orthopaedic‐Traumatologic Sports Medicine
PCL: oosterior cruciate ligament
QoL: quality of life
RDE: remote data entry
SD: standard deviation

## INTRODUCTION

Musculoskeletal sport orthopaedic injuries can affect any joint in the body and are among the most common reasons for consultations with sports physicians and orthopaedic surgeons [16, 29]. These injuries range from acute trauma, such as ligament ruptures and fractures, to chronic overuse conditions that impair athletes and physically active individuals [16, 29]. Advances in surgical techniques, rehabilitation strategies and injury prevention programs have significantly improved treatment outcomes, leading to faster recovery times and enhanced long‐term prognoses [1, 30]. Despite these advancements, the focus of existing literature has been predominantly on the biomechanical and functional aspects of treatment. While the socioeconomic burden of sport orthopaedic injuries has been well documented—demonstrating their impact on healthcare systems, work absenteeism and financial costs—the personal burden experienced by patients remains underrepresented in the literature [25, 36]. Musculoskeletal injuries not only cause physical limitations but also impair various aspects of self‐care, mobility and daily activities, which collectively contribute to a significant reduction in quality of life (QoL). The inability to participate in sports, reduced independence and chronic pain can further lead to psychological distress, including anxiety, depression and decreased overall well‐being [12, 28]. A clear correlation between reduced QoL and psychological burden was already reported in previous literature [34].

Despite the growing recognition of these issues, a comprehensive overview of the effect of musculoskeletal injuries on QoL remains lacking. Additionally, sufficient baseline data for standardised QoL assessments, such as the EuroQoL five dimensions (EQ‐5D) index and EQ‐visual analogue scale (EQ‐VAS), are needed to better understand the magnitude of impairment across different types of injuries and patient populations. Establishing such baseline data is crucial for evaluating treatment success, guiding patient‐centred rehabilitation, and informing healthcare policy.

Thus, the primary objective of this study was to (1) assess the impairment of QoL in patients with musculoskeletal sport orthopaedic injuries prior to surgery using data from the German Arthroscopy Registry (DART). Furthermore, we aimed to (2) establish baseline values for the EQ‐5D Index and EQ‐VAS scores in individuals with musculoskeletal injuries in Germany. By providing these reference data, this study seeks to contribute to a more holistic understanding of the burden of sport orthopaedic injuries beyond biomechanical function, emphasising the importance of patient‐centred outcomes in clinical and research settings.

## MATERIALS AND METHODS

### Study population and data collection

This prospective registry study investigates the preoperative QoL in a nationwide study population based on the DART. All patients with a confirmed sport orthopaedic diagnosis of the shoulder, hip, knee or ankle were included. Patients between November 2017 and January 2025 were included in the analysis. The DART registry was initiated by the German Society for Arthroscopy and Joint‐Surgery (AGA), German Society for Arthroscopy (BVASK) and the Society for Orthopaedic‐Traumatologic Sports Medicine (GOTS) in cooperation the German Society for Orthopaedics and Trauma (DGOU). The aim of this registry is to collect surgery‐ and outcome‐specific data of arthroscopic knee, shoulder, hip and ankle surgeries performed in Germany, Austria and Switzerland. Detailed setup, structure and methods were previously reported by Mueller‐Rath et al. [10, 22]. The registry is a web‐based remote data entry (RDE) system in which the surgeon and patient each complete a survey for a single case. Depending on the treated joint, each case is classified under a single module (shoulder, hip, knee and ankle module). At baseline, the surgeon's section includes mandatory information on patient‐ and joint‐specific characteristics, previous operations (including the contralateral side), all surgical procedures performed on the injured joint (including defect‐specific information) and therapy characteristics. The patient's questionnaire consists of joint‐specific, validated and standardised patient‐reported outcome measures and a joint‐independent QoL assessment (EuroQol five‐dimension questionnaire [EQ‐5D‐3L] including the EQ‐VAS) at first registration and in follow‐up investigations [10, 22]. The EQ‐5D consists of five dimensions—mobility, self‐care, usual activities, pain/discomfort and anxiety/depression. In the three‐level version (EQ‐5D‐3L), each dimension is rated on three severity levels (no problems, some problems, extreme problems), defining a health state that can be converted into a single index value using country‐specific value sets. Index values typically range from values below 0, representing health states worse than death, to 1, representing perfect health. The EQ‐VAS records the patient's self‐rated health on a vertical visual analogue scale from 0 to 100, with 0 corresponding to the worst imaginable health and 100 to the best imaginable health. Written informed consent was gathered from all patients.

**Figure 1:**
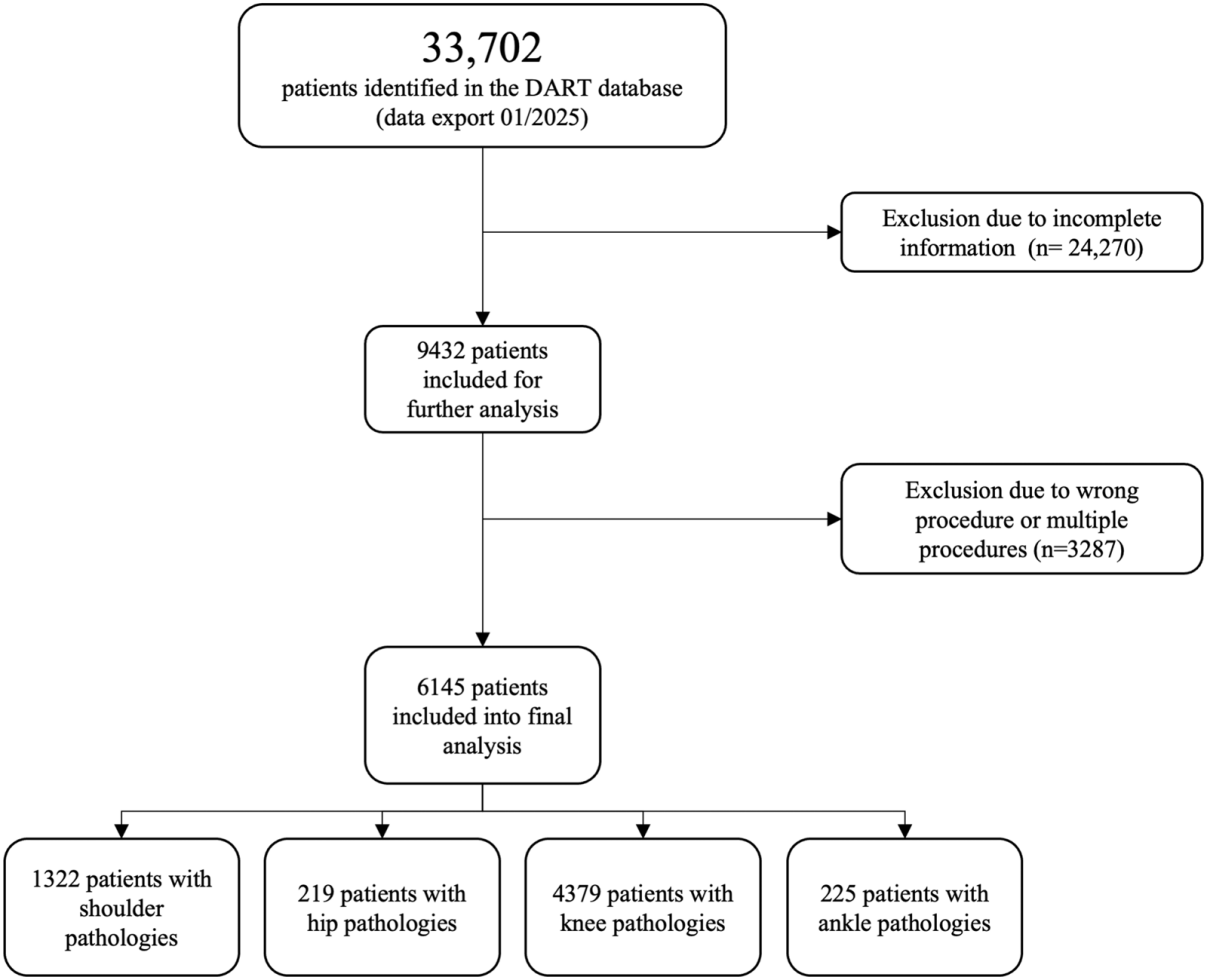
Flowchart of study population. DART, German arthroscopy register.

For confirmed diagnosis before treatment the QoL of patients was assessed by the EQ. 5D‐3L. The diagnosis was identified by categorisation of operating procedure codes (OPS‐codes) (File S1). In diagnosis groups with possible heterogeneity of surgical procedures (e.g., ACL rupture with meniscus injury, shoulder instability), patients were categorised according to the primary diagnosis and all relevant surgical techniques were pooled. As our analysis focused on preoperative QoL, procedure‐specific differentiation was not performed. All accompanying diagnoses related to the primary diagnoses were excluded, therefore only patients with a primary diagnosis and no comorbid conditions were analysed (Figure 1). Patients were also excluded if the available dataset lacked essential baseline information (e.g., missing EQ‐5D or EQ‐VAS values, demographic variables, or key surgical parameters). In addition, cases with incomplete injury documentation or implausible values (e.g., negative symptom duration) were removed prior to analysis. Only primary injuries fulfilling the registry inclusion criteria and with complete preoperative data were retained for statistical evaluation. Meniscus injuries were divided into a future resection and future suture subpopulation due to the different indications and impact on outcomes.

Based on the time‐trade‐off (TTO) value set for Germany by Greiner et al. EQ‐5D states were converted into a single EQ index value [9]. Through this approach values are converted, can represent the societal perspective and can be compared sufficiently international.

All patients in the registry with signed informed consent were included into the data analysis. Diagnosis with a case number <20 patients were excluded from the analysis.

### Statistical analysis and data assessment

Categorial data are expressed as frequency counts (percentages) and continuous data as mean ± standard deviation (SD). IBM SPSS Statistics, version 29.0 was used for data analysis. Graphical visualisation of potential bias and missing data patterns was generated using IBM SPSS Statistics 29.0. One‐way analysis of variance (ANOVA) was used to calculate differences between subpopulations. To explore potential patient‐related predictors of preoperative health status, subgroup analyses were conducted using linear regression models for each pathology separately. Age, gender, body height, body weight and symptom duration until surgery were included as independent variables, with EQ‐5D index as dependent outcomes. Significant predictors (*p* < 0.05) were summarised in a separate table.The DART project is conducted in accordance with the Declaration of Helsinki and registered at germanctr.de (DRKS00012994). The study design was approved by the coordinating institutional review board of the University of Freiburg (No. 624/19) and by the local ethics committees of every participating institution.

## RESULTS

Overall, 9432 patients were included into the data analysis. Most patients in the registry had a pathology of the knee (*n* = 7123; 75.5%) or shoulder (*n* = 1798; 19.1%), while 242 ankle patients (2.6%) and 260 hip patients (2.8%) were included. Meniscus lesions suitable for resection (*n* = 2293) was the most common diagnosis, followed by anterior cruciate ligament (ACL) injuries with meniscus lesion (*n* = 744), isolated rupture of the ACL (*n* = 727) and rotator cuff tears with biceps tendon pathology (*n* = 547). Patient characteristics were summarised in Table 1.

**Table 1:** Anthropometric data of the study population.

Diagnosis	Number	Age in years (min; max)	Height in cm (min; max)	Weight in kg (min; max)	Sex (male/female) number (percentage)	Duration of symptoms in weeks (min; max)
Shoulder						
Subacromial pathology	259	49.6 ± 10.5 (19;75)	173.4 ± 9.1 (155; 194)	81.6 ± 17.6 (36; 140)	124/135 (47.9/52.1)	47.9 ± 37.6 (0; 180)
Shoulder instability	105	29.3 ± 9.4 (18; 57)	178.9 ± 8.2 (156; 196)	82.1 ± 17.2 (56; 145)	81/24 (77.1/22.9)	27.9 ± 55.8 (1; 503)
Rotator cuff tear without biceps tendon pathology	411	54.5 ± 10.1 (21; 72)	173.9 ± 12.5 (158; 241)	84.4 ± 17.4 (57; 120)	242/169 (58.9/41.1)	25.4 ± 22.3 (2; 99)
Rotator cuff tear with biceps tendon pathology	547	58.2 ± 8.8 (33; 76)	173.3 ± 9.8 (154; 187)	84.2 ± 18.3 (52; 145)	337/210 (61.6/38.4)	19.2 ± 20.0 (0; 99)
Hip						
Femoroacetabular impingement	219	38.0 ± 11.4 (18; 62)	176.3 ± 9.7 (152; 216)	78.7 ± 16.7 (47; 137)	123/96 (56.2/43.8)	78.1 ± 95.7 (0; 860)
Knee						
ACL injury without concomitant injury	727	31.4 ± 10.0 (18; 63)	174.7 ± 9.5 (150; 211)	77.4 ± 15.9 (48; 150)	379/348 (52.0/48.0)	14.4 ± 20.8 (0; 300)
ACL injury with meniscus injury	744	34.8 ± 11.7 (18; 65)	176.4 ± 9.6 (104; 213)	82.0 ± 16.1 (47; 150)	467/277 (62.8/37.2)	18.2 ± 42.6 (0; 728)
ACL re‐rupture without concomitant injury	55	32.4 ± 9.8 (19; 59)	176.5 ± 10.5 (153; 213)	81.3 ± 16.5 (50; 120)	37/18 (67.3/32.7)	22.9 ± 34.1 (2; 220)
Meniscus injury with future resection	2293	52.0 ± 11.5 (18; 83)	175.6 ± 11.0 (145; 233)	85.1 ± 17.1 (42; 150)	1363/930 (59.4/40.6)	27.8 ± 42.4 (0; 402)
Meniscus injury with future suture	353	40.5 ± 12.4 (18; 68)	175.1 ± 9.3 (151; 199)	84.1 ± 18.3 (37; 150)	189/164 (53.5/46.5)	25.1 ± 42.5 (0; 459)
PCL rupture	40	37.0 ± 13.9 (18; 67)	176.3 ± 10.9 (154; 195)	85.2 ± 15.7 (60; 121)	24/15 (60.0/40.0)	32.9 ± 31.0 (1; 110)
Patella instability	167	27.9 ± 8.9 (18; 68)	173.1 ± 9.6 (152; 198)	79.8 ± 20.4 (40; 147)	59/108 (35.3/64.7)	37.8 ± 66.7 (1; 700)
Ankle						
Cartilage lesion ankle joint	144	35.4 ± 13.3 (18; 69)	175.4 ± 12.7 (106; 218)	83.9 ± 17.1 (46; 140)	83/61 (57.6/42.4)	58.2 ± 100.6 (0;900)
Lateral ankle instability	81	34.6 ± 12.1 (18; 67)	175.0 ± 8.1 (158; 197)	81.6 ± 14.9 (48; 116)	45/36 (55.6/44.4)	39.7 ± 33.3 (0; 99)

Abbreviations: ACL, anterior cruciate ligament; PCL, posterior cruciate ligament.

### Joint localisation

**Figure 2:**
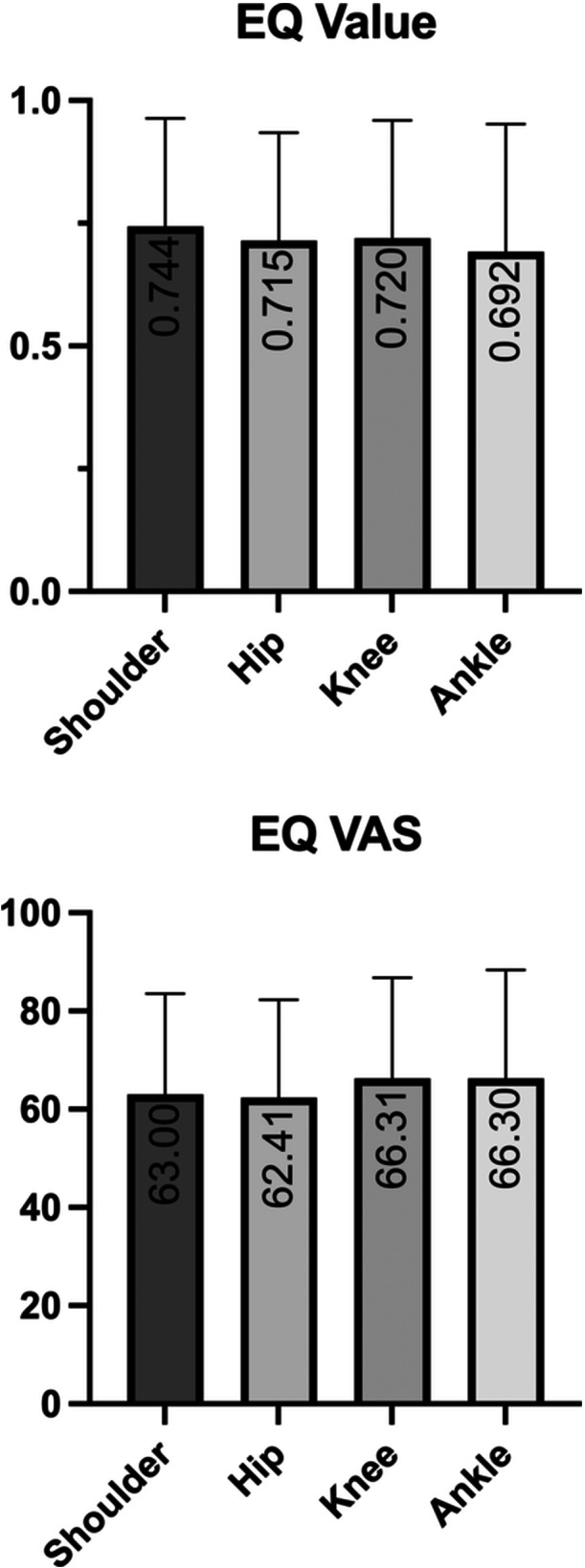
EuroQol (EQ) value based on German time‐trade off value set with 1.0 being the best index and 0 the worst index calculated on all five dimensions of the EQ‐5D and EuroQoL visual analogue scale (EQ‐VAS) with 100 being the best health patients can imagine and 0 the worst health patients can imagine in all shoulder, hip, knee and ankle injuries prior to surgery.

Regarding body region, the highest EQ value was identified in the shoulder joint with 0.744, while patients with a pathology in the ankle joint had the lowest value (0.692). The overall health status was highest in the ankle with an EQ‐VAS with 66.30, while lowest was in the hip with 62.41 (Figure 2). A one‐way ANOVA revealed a statistically significant difference in mean scores between groups for both EQ‐VAS (*F*[3, 9419] = 18.01, *p* < 0.001) and EQ Value (*F*[3, 11,219] = 7.30, *p* < 0.001).

**Figure 3:**
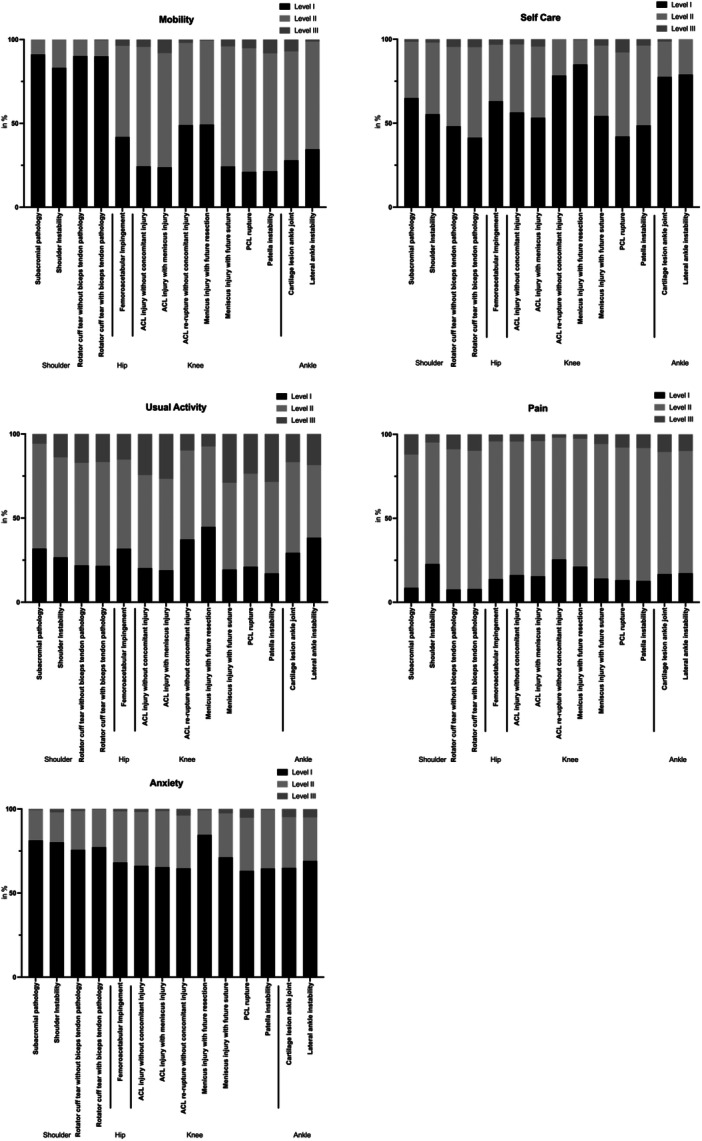
Proportions reporting levels within EQ‐5D‐3L dimensions of all diagnosis in shoulder, hip, knee and ankle injuries (Level I = no problems; Level II = some problems; Level III = extreme problems) ACL, anterior cruciate ligament; PCL, posterior cruciate ligament.

In the analysis of separate dimensions of the EQ‐5D shoulder injuries demonstrated a high proportion (89.8%) of *no problems* (level I) for mobility, while for hip (57.2%), knee (61.5%) and ankle injuries (62.9%) over half of patients reported here *some* (level II) or *severe problems* (level III). Self‐care was mostly impaired in shoulder patients (49.9%), in particular in patients with rotator cuff tear (58.6%) with biceps tendon pathology, while in hip (63.8%), knee (68.5%) and ankle injuries (77.9%) *no problems* were indicated predominantly. The usual activity was in particular in shoulder patients impaired (76.6%). In all joints and injuries pain was present at levels between *some problems* and *extreme problems*. Anxiety, however, was mainly reported as *no problem* for all diagnosis (Figure 3).

### Quality of Life

**Figure 4:**
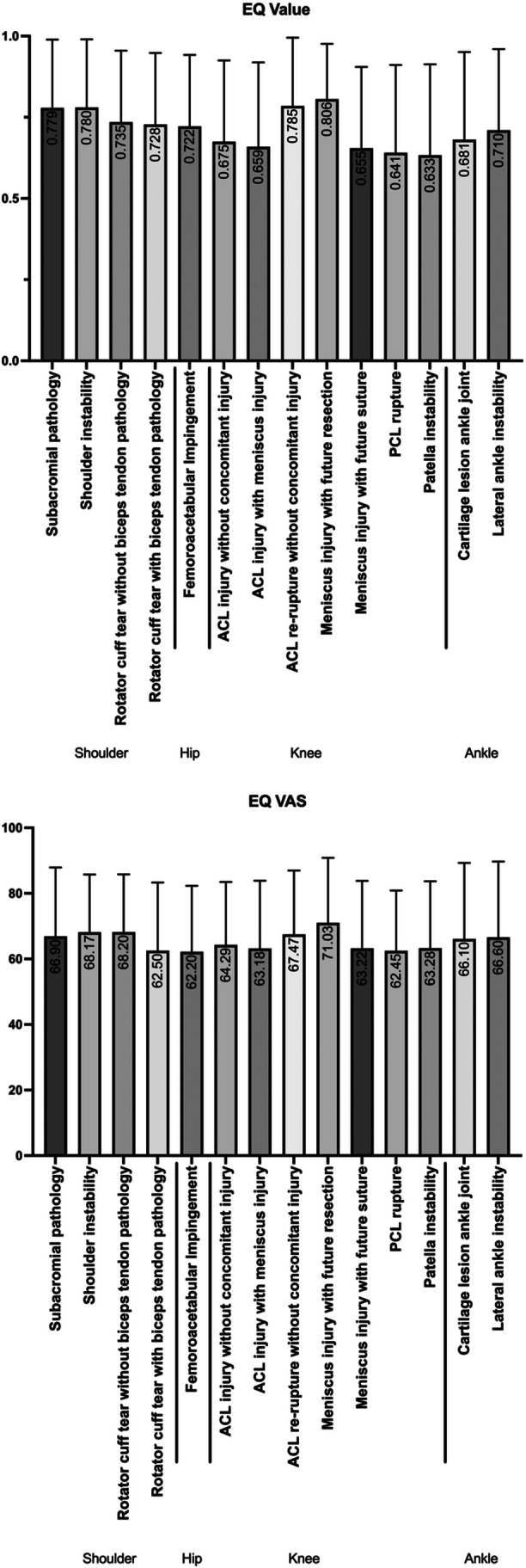
EuroQol (EQ) index value and EuroQoL visual analogue scale (EQ‐VAS) with corresponding standard derivation for all analysed diagnosis in shoulder, hip, knee and ankle patients. ACL, anterior cruciate ligament; PCL, posterior cruciate ligament.

The highest EQ index value was reported in meniscus lesion with future resection (0.806) and ACL re‐ruptures (0.785), while lowest were identified in patella instability (0.633) and rupture of the PCL (0.641). Highest EQ‐VAS was found for meniscus lesion with future resection with 71.03 and rotator cuff tear without biceps tendon pathology (68.20). The worst overall health was reported for rotator cuff tear with biceps tendon pathology (62.50), PCL rupture (62.45) and femoroacetabular impingement (FAI) (62.20) (Figure 4). A one‐way ANOVA demonstrated significant differences in EQ Value among shoulder pathology subgroups (*F*[3, 460] = 15.79, *p* < 0.001) and among knee‐related pathology subgroups (*F*[6, 4372] = 84.07, *p* < 0.001), indicating that health‐related QoL varied substantially across specific diagnostic categories within each joint.

Subgroup analyses across all pathologies revealed only few significant associations between patient characteristics and preoperative EQ‐5D values. Negative predictors included higher body weight (shoulder instability, isolated ACL, meniscus resection) and older age (ACL with meniscus, meniscus procedures). Taller body height was consistently associated with better health state values in some groups (shoulder instability, ankle cartilage, meniscus resection). Symptom duration until surgery showed inconsistent effects, being positively associated with EQ‐5D in isolated ACL, but negatively in revision ACL. For most other pathologies, no significant predictors were identified (Table 2).

**Table 2:** Significant predictors of preoperative EQ‐5D health state value across different pathologies.

Pathology	Predictor	*B* (Unstandardised)	*p*‐Value	Interpretation
Shoulder instability	Height	0.031	*p* = 0.032	Taller = better
Shoulder instability	Weight	−0.031	*p* = 0.042	Heavier = worse
Cartilage lesion ankle joint	Height	0.004	*p* = 0.023	Taller = better
ACL injury without concomitant injury	Symptom duration	0.002	*p* < 0.001	Longer = better
ACL injury without concomitant injury	Weight	−0.002	*p* = 0.034	Heavier = worse
ACL injury with meniscus injury	Age	0.002	*p* = 0.020	Older = worse
ACL re‐rupture without concomitant injury	Symptom duration	−0.002	*p* = 0.005	Longer = worse
Meniscus injury with future resection	Weight	−0.001	*p* = 0.026	Heavier = worse
Meniscus injury with future resection	Height	0.001	*p = *0.002	Taller = better
Meniscus injury with future resection	Age	0.001	*p* = 0.012	Older = worse
Meniscus injury with future suture	Age	0.002	*p* = 0.040	Older = worse

Abbreviation: ACL, anterior cruciate ligament.

## DISCUSSION

Main finding of this analysis of the DART on over 9400 patient was the detection of major impairment of QoL and general health status for musculoskeletal sport orthopaedic injuries prior to surgery. Lowest QoL by EQ‐5D index value was identified in patella instability, PCL rupture, ACL rupture with meniscus injury and meniscus injuries with future suture. The lowest EQ‐VAS was prevalent in FAI, PCL rupture, patella instability, rotator cuff tear with biceps tendon pathology and ACL rupture with meniscus injury.

Reduction of QoL and correlating psychological burden on athletes and patients is an issue in all fields of medicine. In orthopaedic surgery, mental health status and corresponding QoL was neglected and main focus was set on surgical therapy and rehabilitation [12]. For the first time this work demonstrates the impairment of patients’ QoL in several sports orthopaedic injuries with their diagnosis prior any surgical treatment.

Previous reports on QoL in sport orthopaedics focus on changes to report efforts of an treatment or rehabilitation protocol. However, similar results of EQ‐5D index values and EQ‐VAS were identified for injuries in the shoulder [13, 23, 26], hip [11, 35, 40], knee [14, 17, 27] and ankle joint [18, 37] in these publications.

In shoulder pathologies Nicholson et al. demonstrated in rotator cuff injuries an EQ. 5D value of 0.54 prior to surgery [23]. In another investigation by Marks et al. in Germany a EQ. 5D value of 0.71 was reported prior to rotator cuff repair [20]. This is similar to our results of the DART with a value of 0.735 in rotator cuff injuries without biceps pathology and 0.728 with biceps pathology. For patients with pathologies of the hip joint Holleyman et al. showed for FAI a value of 0.52 prior to surgery and an EQ‐VAS of 66.6 [11]. In our population for FAI the EQ‐VAS of 62.2 was slightly inferior to the cohort of Holleyman while the EQ value of 0.722 was superior. In patients with meniscus injuries Lee et al. reported an EQ value of 0.55 with admission to the hospital [17]. In the DART population, an EQ value of 0.65 was slightly superior. In patients with chronic lateral instability of the ankle joint, Vignaraja et al demonstrated a preoperative EQ value of 0.607 and an EQ‐VAS of 69.9 [37]. The EQ‐VAS of all patients included in our DART collective was similar with 66.6, while the EQ value was superior with 0.71.

Sports orthopaedic injuries lead to several problems and impairments in all dimensions of QoL. In particular, usual activity and pain were identified for all injuries as a relevant factor. Further mobility was an issue for patients with injuries of the lower extremity, while patients with injuries of the shoulder had most limitations in self‐care. The reduction of mobility in lower extremity injuries is well known due to immobilisation in emergency care and in several chronic injuries [6]. Interesting, the reduction in QoL is similar in upper and lower extremity injuries. QoL is not only impaired immediately after the injury, but also several years after surgical treatment. For ACL injuries even after 5 years an reduction compared to the population was identified in a previous meta‐analysis [7].

When the results of QoL in patient with musculoskeletal orthopaedic injuries are compared with those of other nonsport‐orthopaedic diseases, a similar impairment can be identified. In our study, EQ index values ranging from 0.633 to 0.806 were reported for various injuries in the DART. This reduction in QoL was comparable to that experienced by patients, who had sustained polytrauma with an injury severity score (ISS) over 16 at their 6‐month follow‐up (EQ index value: 0.71) [8]. Similar results were demonstrated for the general health status, as measured with the EQ‐VAS, which was in our population between 57.09 and 71.03, whereas in patients after polytrauma, it was 62.0 at their 6‐month follow‐up [8]. Similar reductions in QoL were also reported in previous investigations of pelvic ring fractures, with EQ‐5D index values of 0.61 and 0.74 at 6 weeks and 3 months after the incident, respectively [2].

Interestingly, however, these effects of a similar reduction of QoL were also identified in cardiology, in patients who suffered myocardial infarctions, with an EQ index value of 0.73 and an EQ‐VAS of 75 [24]. In oncology, patients with colorectal cancer had an EQ index value of 0.82 on average, which was better than all patients in our registry, and an EQ‐VAS of 62.05, similar to our sports orthopaedic data [4]. Similar effects were found in lung cancer patients with an EQ index value of 0.74 after surgery [15] and a mean health state score of EQ‐VAS 56.7 [3]. These results, alongside comparisons to oncological and cardiological diseases, underline the relevance of QoL in sports orthopaedic injuries and demonstrate the severe impairment experienced by patients. This reduction in QoL and in general health status, accompanied by impairment of mental health, should not be neglected. While musculoskeletal and sports orthopaedic conditions are often considered secondary in health services research compared to high‐profile disciplines such as cardiology or oncology, our findings demonstrate a comparable reduction in QoL. This underscores the substantial burden of these conditions and supports the need for stronger integration of orthopaedic outcomes in health policy and resource allocation debates.

While the EQ‐5D index value is calculated based on five dimensions—mobility, self‐care, usual activity, pain and anxiety—due to sport orthopaedic injuries and immobilisation by casts or orthosis prior to surgical treatment or due to painful limitations of daily tasks, the reduction in the index value can be explained. However, the psychological aspect and the patient's mental health status must also be considered for further treatment and the healing process [28]. Injuries are known to have also a negative effect on mental health and impaired mental health can constrain further healing processes [32]. Furthermore, pre‐existing psychological factors are associated with the outcome of musculoskeletal diseases and should always be taken into consideration [5, 21, 33].

### Clinical implications

Based on the results of this study, physicians and health care practitioners should also be made aware of issues relating to impairment in QoL and patient's mental health. Confidence in the rehabilitation process is an essential factor in a successful return to sports and should be considered alongside physical therapy after any treatment [31, 39]. The implementation of biopsychological interventions, or at least the measurement of psychological burden using patient‐related outcome measures (e.g., the ACL Return to Sport after Injury Scale for ACL injuries), is recommended [31, 33, 38]. Further, these results underline the importance of sports orthopaedic injuries and the relevance of prevention in sports. Several programs are approved to reduce injury burden with economic cost savings, respectively [19].

### Limitations

In addition to the advantages of this study, some limitations due to its design must also be reported. Patients are included in the registry prior to a surgical procedure. Therefore, only patients requiring surgical treatment are included. Patients with minor issues or minor manifestations of the reported sports orthopaedic injury are excluded from this study. Patients who have undergone previous conservative treatment and switched to surgical treatment are also included. The QoL of these patients may be worse due to failed conservative treatment and prolonged therapy. This study excluded patients with multiple pathologies in one joint and only common combinations (e.g., ACL and meniscus) were included in the analysis. However, several minor pathologies often occur in an injured joint and can affect QoL. A limitation of this study is the use of the EQ‐5D‐3L, which is known to exhibit ceiling effects, particularly in younger and healthier populations. As a result, subtle but clinically relevant impairments may not be fully captured. The EQ‐5D‐5L reduces these ceiling effects and improves sensitivity; however, we used the EQ‐5D‐3L from the beginning of the registry and could not change the instrument to maintain consistency in longitudinal data.

## CONCLUSION

Sport orthopaedic injuries represent a substantial burden not only in terms of physical impairment but also with respect to patients' overall QoL and perceived health. These findings from the DART highlight the importance of adopting a more holistic treatment approach that incorporates both physical and psychological dimensions of recovery. Greater attention to mental health and patient‐reported outcomes may improve long‐term treatment success, and future research should explore biopsychological interventions and structured QoL monitoring as integral parts of clinical care.

## AUTHOR CONTRIBUTIONS

**Dominik Szymski**: Formal analysis; investigation; visualisation; writing—original draft. **Christoph Lutter**: Conceptualisation; methodology; writing—review and editing. **Sina Graeber**: Software; formal analysis; writing—review and editing. **Alexander‐Stephan Henze**: Investigation, writing—review and editing. **Maximilian Hinz**: Data curation; methodology; resources; writing—review and editing. **Anja Hirschmueller**: Validation; investigation; writing—review and editing. **Thomas Niethammer**: Resources; investigation; writing—review and editing. **Ralf Müller Rath**: Project administration; methodology; validation; writing—review and editing. **Thomas Tischer**: Supervision; conceptualisation; writing—original draft. All authors read and approved the final manuscript.

## CONFLICT OF INTEREST STATEMENT

The authors declare no conflicts of interest.

## ETHICS STATEMENT

The study was approved by the Ethics Committee of the University of Freiburg (ID: 624/19). Informed consent was obtained from all participants.

## Supporting information

Supplement 1: OPS‐procedure codes and corresponding diagnosis
Supplement 2: Mean values of dimensions of the EQ‐5D‐3L and the EQ Value in shoulder injuries
Supplement 3: Number and proportion in dimensions of the EQ‐5D‐3L in shoulder injuries (Level I = no problems; Level II = some problems; Level III = extreme problems)
Supplement 4: Mean values of dimensions of the EQ‐5D‐3L and the EQ Value in hip injuries
Supplement 5: Number and proportion in dimensions of the EQ‐5D‐3L in hip injuries (Level I = no problems; Level II = some problems; Level III = extreme problems)
Supplement 6: Mean values of dimensions of the EQ‐5D‐3L and the EQ Value in knee injuries
Supplement 7: Number and proportion in dimensions of the EQ‐5D‐3L in knee injuries (Level I = no problems; Level II = some problems; Level III = extreme problems)
Supplement 8: Mean values of dimensions of the EQ‐5D‐3L and the EQ Value in ankle injuries
Supplement 9: Number and proportion in dimensions of the EQ‐5D‐3L in ankle injuries (Level I = no problems; Level II = some problems; Level III = extreme problems).

## Data Availability

Data are available upon request.
