# Supplementary material for: Impairment in quality of life with different sport orthopaedic musculoskeletal injuries—A comprehensive analysis of the German arthroscopy register
Source: Knee Surg Sports Traumatol Arthrosc. 2025 Nov 18;34(6):2067–77. doi: 10.1002/ksa.70205 (PMC13266943; doi:10.1002/ksa.70205)
Supplement: Supplementary file 1 — Supplement 1: OPS‐procedure codes and corresponding diagnosis Supplement 2: Mean values of dimensions of the EQ‐5D‐3L and the EQ Value in shoulder injuries Supplement 3: Number and proportion in dimensions of the EQ‐5D‐3L in shoulder injuries (Level I = no problems; Level II = some problems; Level III = extreme problems) Supplement 4: Mean values of dimensions of the EQ‐5D‐3L and the EQ Value in hip injuries Supplement 5: Number and proportion in dimensions of the EQ‐5D‐3L in hip injuries (Level I = no problems; Level II = some problems; Level III = extreme problems) Supplement 6: Mean values of dimensions of the EQ‐5D‐3L and the EQ Value in knee injuries Supplement 7: Number and proportion in dimensions of the EQ‐5D‐3L in knee injuries (Level I = no problems; Level II = some problems; Level III = extreme problems) Supplement 8: Mean values of dimensions of the EQ‐5D‐3L and the EQ Value in ankle injuries Supplement 9: Number and proportion in dimensions of the EQ‐5D‐3L in ankle injuries (Level I = no problems; Level II = some problems; Level III = extreme problems). [file KSA-34-2067-s001.docx]

**Supplement:
Supplement 1:** OPS-procedure codes and corresponding diagnosis

| **Diagnosis** | **OPS-Codes** |
| --- | --- |
| *Shoulder* | |
| Subacromial pathology | 5-814.3, 5-810.50, 5-814.c, 5-810.20, 5-782.b0, 5-814.b |
| Biceps tendon pathology | 5-814.7, 5-814.9, 5-814.0, 5-814.8 |
| Rotator cuff tear | 5-814.4, 5-819.10, 5-814.6, 5-814.e |
| Shoulder instability | 5-814.0, 5-814.2, 5-814.5, 5-784.dz, 5-784.fz, 5-805.2 |
| *Hip* | |
| Femoroacetabular impingement | 5-812.eg, 5-812.kg, 5-816.0, 5-816.1, 5-816.2, 5-782.ad, 5-782.ae, 5-782.bd, 5-782.be, 5-782.1d, 5-782.1e, 5-782.1f |
| *Knee* | |
| ACL injury | 5-813.4, 5-813.3, 5-813.g, 5-813.0, 5-813.j, 5-813.2 |
| ACL re-rupture | 5-815.21, 5-815.31, 5-815.20, 5-815.23, 5-780.6k, 5-780.6h, 5-810.3h, 5-783.0, 5-784.0k, 5-784.ch, 5-784.70, 5-784.eh |
| Meniscus injury with future resection | 5-812.6, 5-812.5 |
| Meniscus injury with future suture | 5-812.7 |
| PCL rupture | 5-813.7, 5-813.6, 5-813.h, 5-813.1, 5-813.2 |
| Patella instability | 5-813.9, 5-813.a, 5-804.8, 5-804.3, 5-781.8h, 5-804.8,  5-802.4, 5-804.7, 5-856.18, 5-804.0 |
| *Ankle* | |
| Cartilage lesion ankle joint | 5-812.ek, 5-812.fk, 5-812.3k, 5-812.hk, 5-812.gk, 5-812.9k, 5-801.kk, 5.801.bk |
| Lateral ankle instability | 5-819.4, 5-806.c, 5-806.4, 5-806.5, 5-806.7, 5-806.6, 5-806.9 |

**Supplement 2:** Mean values of dimensions of the EQ-5D-3L and the EQ Value in shoulder injuries

|  | Mobility | Self-Care | Usual activities | Pain/  Discomfort | Anxiety/  Depression | EQ VAS | EQ Value |
| --- | --- | --- | --- | --- | --- | --- | --- |
| Subacromial pathology | 1.09 ± 0.29 | 1.37 ± 0.51 | 1.74 ± 0.56 | 2.04 ± 0.45 | 1.19 ± 0.42 | 66.9 ± 21.0 | 0.779 ± 0.21 |
| Shoulder Instability | 1.17 ± 0.37 | 1.47 ± 0.54 | 1.87 ± 0.63 | 1.82 ± 0.50 | 1.22 ± 0.46 | 68.2 ± 17.6 | 0.780 ± 0.21 |
| Rotator cuff tear without biceps tendon pathology | 1.10 ± 0.29 | 1.57 ± 0.58 | 1.95 ± 0.62 | 2.01 ± 0.40 | 1.26 ± 0.46 | 62.5 ± 20.8 | 0.735 ± 0.22 |
| Rotator cuff tear with biceps tendon pathology | 1.10 ± 0.31 | 1.63 ± 0.57 | 1.95 ± 0.61 | 2.02 ± 0.42 | 1.23 ± 0.43 | 61.5 ± 21.1 | 0.728 ± 0.22 |
| Total | 1.10 ± 0.31 | 1.53 ± 0.56 | 1.91 ± 0.61 | 2.02 ± 0.42 | 1.25 ± 0.46 | 63.0 ± 20.5 | 0.744 ± 0.22 |

**Supplement 3:** Number and proportion in dimensions of the EQ-5D-3L in shoulder injuries (Level I = no problems; Level II = some problems; Level III = extreme problems)

|  |  | Subacromial pathology | Shoulder Instability | Rotator cuff tear without biceps tendon pathology | Rotator cuff tear with biceps tendon pathology | Total |
| --- | --- | --- | --- | --- | --- | --- |
| Mobility | Level I | 234 (91.1) | 84 (83.2) | 367 (90.2) | 479 (90.0) | 1586 (89.8) |
|  | Level II | 23 (8.9) | 17 (16.8) | 40 (9.8) | 51 (9.6) | 177 (10.0) |
|  | Level III | 0 (0.0) | 0 (0.0) | 0 (0.0) | 2 (0.4) | 3 (0.2) |
| Self-Care | Level I | 167 (65.0) | 56 (55.4) | 196 (48.2) | 220 (41.4) | 885 (50.1) |
|  | Level II | 86 (33.5) | 43 (42.6) | 192 (47.2) | 287 (53.9) | 821 (46.5) |
|  | Level III | 4 (1.6) | 2 (2.0) | 19 (4.7) | 25 (4.7) | 60 (3.4) |
| Usual activities | Level I | 82 (31.9) | 27 (26.7) | 89 (21.9) | 115 (21.6) | 413 (23.4) |
|  | Level II | 160 (62.3) | 60 (59.4) | 248 (60.9) | 328 (61.7) | 1104 (62.5) |
|  | Level III | 15 (5.8) | 14 (13.9) | 70 (17.2) | 89 (16.7) | 249 (14.1) |
| Pain/  Discomfort | Level I | 22 (8.6) | 23 (22.8) | 31 (7.6) | 42 (7.9) | 142 (8.0) |
|  | Level II | 204 (79.4) | 73 (72.3) | 340 (83.5) | 438 (82.3) | 1452 (82.2) |
|  | Level III | 31 (12.1) | 5 (5.0) | 36 (8.8) | 52 (9.8) | 172 (9.7) |
| Anxiety/  Depression | Level I | 209 (81.3) | 81 (80.2) | 308 (75.7) | 411 (77.3) | 1348 (76.3) |
|  | Level II | 46 (17.9) | 18 (17.8) | 94 (23.1) | 118 (22.2) | 398 (22.5) |
|  | Level III | 2 (0.8) | 2 (2.0) | 5 (1.2) | 3 (0.6) | 20 (1.1) |

**Supplement 4:** Mean values of dimensions of the EQ-5D-3L and the EQ Value in hip injuries

|  | Mobility | Self-Care | Usual activities | Pain/  Discomfort | Anxiety/  Depression | EQ VAS | EQ Value |
| --- | --- | --- | --- | --- | --- | --- | --- |
| Femoroacetabular Impingement | 1.62 ± 0.56 | 1.40 ± 0.55 | 1.83  ± 0.67 | 1.90 ± 0.41 | 1.33 ± 0.50 | 62.2 ± 20.1 | 0.722 ± 0.22 |
| Total | 1.64 ± 0.55 | 1.39 ± 0.54 | 1.85 ± 0.67 | 1.93 ± 0.41 | 1.33 ± 0.50 | 62.41 ± 19.9 | 0.715 ± 0.22 |

**Supplement 5:** Number and proportion in dimensions of the EQ-5D-3L in hip injuries (Level I = no problems; Level II = some problems; Level III = extreme problems)

|  |  | Femoroacetabular Impingement | Total |
| --- | --- | --- | --- |
| Mobility | Level I | 91 (41.9) | 101 (39.3) |
|  | Level II | 118 (54.4) | 147 (57.2) |
|  | Level III | 8 (3.7) | 9 (3.5) |
| Self-Care | Level I | 137 (63.1) | 164 (63.8) |
|  | Level II | 73 (33.6) | 86 (33.5) |
|  | Level III | 7 (3.2) | 7 (2.7) |
| Usual activities | Level I | 69 (31.8) | 79 (30.7) |
|  | Level II | 115 (53.0) | 138 (53.7) |
|  | Level III | 33 (15.2) | 40 (15.6) |
| Pain/  Discomfort | Level I | 30 (13.8) | 32 (12.5) |
|  | Level II | 178 (82.0) | 212 (82.5) |
|  | Level III | 9 (4.1) | 13 (5.1) |
| Anxiety/  Depression | Level I | 148 (68.2) | 176 (68.5) |
|  | Level II | 66 (30.4) | 77 (30.0) |
|  | Level III | 3 (1.4) | 4 (1.6) |

**Supplement 6:** Mean values of dimensions of the EQ-5D-3L and the EQ Value in knee injuries

|  | Mobility | Self-Care | Usual activities | Pain/  Discomfort | Anxiety/  Depression | EQ VAS | EQ Value |
| --- | --- | --- | --- | --- | --- | --- | --- |
| ACL injury without concomitant injury | 1.80 ± 0.50 | 1.47 ± 0.56 | 2.04 ± 0.67 | 1.88 ± 0.44 | 1.36 ± 0.52 | 64.29 ± 19.2 | 0.675 ± 0.25 |
| ACL injury with meniscus injury | 1.84 ± 0.54 | 1.51 ± 0.58 | 2.08 ± 0.67 | 1.88 ± 0.43 | 1.36 ± 0.51 | 63.18 ± 20.7 | 0.659 ± 0.26 |
| ACL re-rupture without concomitant injury | 1.53 ± 0.54 | 1.22 ± 0.42 | 1.73 ± 0.64 | 1.76 ± 0.47 | 1.39 ± 0.57 | 67.47 ± 19.5 | 0.785 ± 0.21 |
| Meniscus injury with furutre resection | 1.51 ± 0.51 | 1.16 ± 0.38 | 1.63 ± 0.62 | 1.81 ± 0.45 | 1.16 ± 0.39 | 71.03 ± 19.8 | 0.806 ± 0.17 |
| Meniscus injury with future suture | 1.80 ± 0.49 | 1.50 ± 0.57 | 2.10 ± 0.69 | 1.92 ± 0.44 | 1.31 ± 0.52 | 63.22 ± 20.6 | 0.655 ± 0.25 |
| PCL rupture | 1.84 ± 0.50 | 1.66 ± 0.63 | 2.03 ± 0.68 | 1.95 ± 0.46 | 1.42 ± 0.60 | 62.45 ± 18.4 | 0.641 ± 0.27 |
| Patella instability | 1.87 ± 0.53 | 1.55 ± 0.57 | 2.11 ± 0.67 | 1.96 ± 0.45 | 1.36 ± 0.59 | 63.28 ± 20.4 | 0.633 ± 0.28 |
| Total | 1.69 ± 0.54 | 1.34 ± 0.52 | 1.87 ± 0.68 | 1.88 ± 0.45 | 1.27 ± 0.48 | 66.31 ± 20.5 | 0.720 ± 0.24 |

**Supplement 7:** Number and proportion in dimensions of the EQ-5D-3L in knee injuries (Level I = no problems; Level II = some problems; Level III = extreme problems)

|  |  | ACL injury without concomitant injury | ACL injury with meniscus injury | ACL re-rupture without concomitant injury | Meniscus injury with future resection | Meniscus injury with future suture | PCL rupture | Patella instability | Total |
| --- | --- | --- | --- | --- | --- | --- | --- | --- | --- |
| Mobility | Level I | 172 (24.4) | 173 (23.8) | 25 (49.0) | 1099 (49.3) | 83 (24.3) | 8 (21.1) | 34 (21.5) | 2412 (34.9) |
|  | Level II | 501 (71.2) | 496 (68.1) | 25 (49.0) | 1117 (50.1) | 244 (71.6) | 28 (73.7) | 111 (70.3) | 4251 (61.5) |
|  | Level III | 31 (4.4) | 59 (8.1) | 1 (2.0) | 12 (0.5) | 14 (4.1) | 2 (5.3) | 13 (8.2) | 248 (3.6) |
| Self-Care | Level I | 397 (56.4) | 388 (53.3) | 40 (78.4) | 1893 (85.0) | 185 (54.3) | 16 (42.1) | 77 (48.7) | 4732 (68.5) |
|  | Level II | 285 (40.5) | 308 (42.3) | 11 (21.6) | 323 (14.5) | 143 (41.9) | 19 (50.0) | 75 (47.5) | 2022 (29.3) |
|  | Level III | 22 (3.1) | 32 (4.4) | 0 (0.0) | 12 (0.5) | 13 (3.8) | 3 (7.9) | 6 (3.8) | 157 (2.3) |
| Usual activities | Level I | 143 (20.3) | 138 (19.0) | 19 (37.3) | 998 (44.8) | 66 (19.4) | 8 (21.1) | 27 (17.1) | 2125 (30.7) |
|  | Level II | 389 (55.3) | 395 (54.3) | 27 (52.9) | 1064 (47.8) | 176 (51.6) | 21 (55.3) | 86 (54.4) | 3574 (51.7) |
|  | Level III | 172 (24.4) | 195 (26.8) | 5 (9.8) | 166 (7.5) | 99 (29.0) | 9 (23.7) | 45 (28.5) | 1212 (17.5) |
| Pain/  Discomfort | Level I | 114 (16.2) | 113 (15.5) | 13 (25.5) | 473 (21.2) | 48 (14.1) | 5 (13.2) | 20 (12.7) | 1181 (17.1) |
|  | Level II | 560 (79.5) | 586 (80.5) | 37 (72.5) | 1697 (76.2) | 273 (80.1) | 30 (78.9) | 125 (79.1) | 5412 (78.3) |
|  | Level III | 30 (4.3) | 29 (4.0) | 1 (2.0) | 58 (2.6) | 20 (5.9) | 3 (7.9) | 13 (8.2) | 318 (4.6) |
| Anxiety/  Depression | Level I | 466 (66.2) | 476 (65.4) | 33 (64.7) | 1884 (84.6) | 243 (71.3) | 24 (63.2) | 102 (64.6) | 5142 (74.4) |
|  | Level II | 225 (32.0) | 243 (33.4) | 16 (31.4) | 325 (14.6) | 89 (26.1) | 12 (31.6) | 55 (34.8) | 1648 (23.8) |
|  | Level III | 13 (1.8) | 9 (1.2) | 2 (3.9) | 18 (0.8) | 9 (2.6) | 2 (5.3) | 1 (0.6) | 120 (1.7) |

**Supplement 8:** Mean values of dimensions of the EQ-5D-3L and the EQ Value in ankle injuries

|  | Mobility | Self-Care | Usual activities | Pain/  Discomfort | Anxiety/  Depression | EQ VAS | EQ Value |
| --- | --- | --- | --- | --- | --- | --- | --- |
| Cartilage lesion ankle joint | 1.79 ± 0.56 | 1.24 ± 0.46 | 1.87 ± 0.67 | 1.94 ± 0.52 | 1.40 ± 0.58 | 66.1 ± 23.2 | 0.681 ± 0.27 |
| Lateral ankle instability | 1.67 ± 0.50 | 1.21 ± 0.41 | 1.80 ± 0.73 | 1.93 ± 0.52 | 1.36 ± 0.58 | 66.6 ± 23.1 | 0.710 ± 0.25 |
| Total | 1.74 ± 0.55 | 1.24 ± 0.46 | 1.87 ± 0.66 | 1.96 ± 0.50 | 1.36 ± 0.57 | 66.3 ± 22.1 | 0.692 ± 0.26 |

**Supplement 9:** Number and proportion in dimensions of the EQ-5D-3L in ankle injuries (Level I = no problems; Level II = some problems; Level III = extreme problems)

|  |  | Cartilage lesion ankle joint | Lateral ankle instability | Total |
| --- | --- | --- | --- | --- |
| Mobility | Level I | 40 (28.0) | 28 (34.6) | 76 (31.7) |
|  | Level II | 93 (65.0) | 52 (64.2) | 151 (62.9) |
|  | Level III | 10 (7.0) | 1 (1.2) | 13 (5.4) |
| Self-Care | Level I | 111 (77.6) | 64 (79.0) | 187 (77.9) |
|  | Level II | 30 (21.0) | 17 (21.0) | 49 (20.4) |
|  | Level III | 2 (1.4) | 0 (0.0) | 4 (1.7) |
| Usual activities | Level I | 42 (29.4) | 31 (38.3) | 69 (28.7) |
|  | Level II | 77 (53.8) | 35 (43.2) | 132 (55.0) |
|  | Level III | 24 (16.8) | 15 (18.5) | 39 (16.3) |
| Pain/  Discomfort | Level I | 24 (16.8) | 14 (17.3) | 35 (14.6) |
|  | Level II | 104 (72.7) | 59 (72.8) | 180 (75.0) |
|  | Level III | 15 (10.5) | 8 (9.9) | 25 (10.4) |
| Anxiety/  Depression | Level I | 93 (65.0) | 56 (69.1) | 165 (68.8) |
|  | Level II | 43 (30.1) | 21 (25.9) | 64 (26.7) |
|  | Level III | 7 (4.9) | 4 (4.9) | 11 (4.6) |
